# Anthelmintic pyrvinium pamoate blocks Wnt/β-catenin and induces apoptosis in multiple myeloma cells

**DOI:** 10.3892/ol.2020.12110

**Published:** 2020-09-15

**Authors:** Fang Xu, Yingjie Zhu, Yuhong Lu, Zhi Yu, Jun Zhong, Yangqiu Li, Jingxuan Pan

Oncol Lett 15: 5871-5878, 2018; DOI: 10.3892/ol.2018.8006

Following the publication of the above article, the authors have realized that there was one misplaced image in Fig. 1D (the immunoblot shown for active caspase 3), and also a misplaced image in Fig. 3C (the LP1 0–48 h data for caspase 9). The corrected versions of Figs. 1 and 3 are presented opposite and on the next page, showing the correct data as specified. Note that these errors did not impact the conclusions of the manuscript, due to the similarity of the correct data to the misplaced data. In addition, the acknowledgements were not accurately described. The Acknowledgements section should have been written as follows: “The present study was supported by Research Fund for Teachers in Jinan University (to J.P.)”. All the authors agree with this corrigendum. We would like to thank the Editor of *Oncology Letters* for given us the opportunity to publish this corrigendum, and sincerely apologize to the readers for our inadvertent errors that were introduced during the preparation and revision of manuscript.

## Figures and Tables

**Figure 1.**
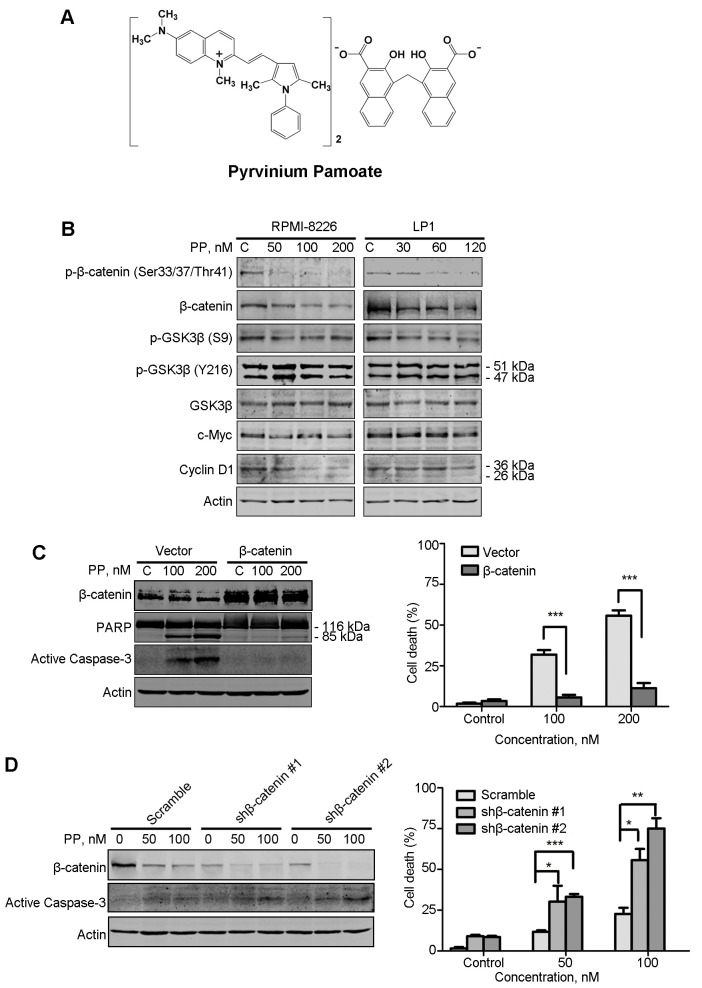
PP blocks Wnt/β-catenin signaling. (A) Molecular structure of PP. (B) RPMI-8226 and LP1 cells were treated with PP at the indicated concentrations for 48 h, and western blot analysis was performed with whole cell lysates. RPMI-8226 cells were transfected with (C) plasmids (empty vector or β-catenin) or (D) shRNAs (scramble or β-catenin targeting). The cells were treated with the indicated concentration of PP, cell lysates were subjected to western blot analysis and the degree of cell death was determined using a trypan blue exclusion assay. *P<0.05, **P<0.01, ***P<0.001; β-catenin vs. empty vector, shβ-catenin #1 or shβ-catenin #2 vs. Scramble, respectively. shRNA, short hairpin RNA; PP, pyrvinium pamoate; p-GSK3β, phosphorylated glycogen synthase kinase-3β; PARP, poly(ADP ribose) polymerase.

**Figure 3.**
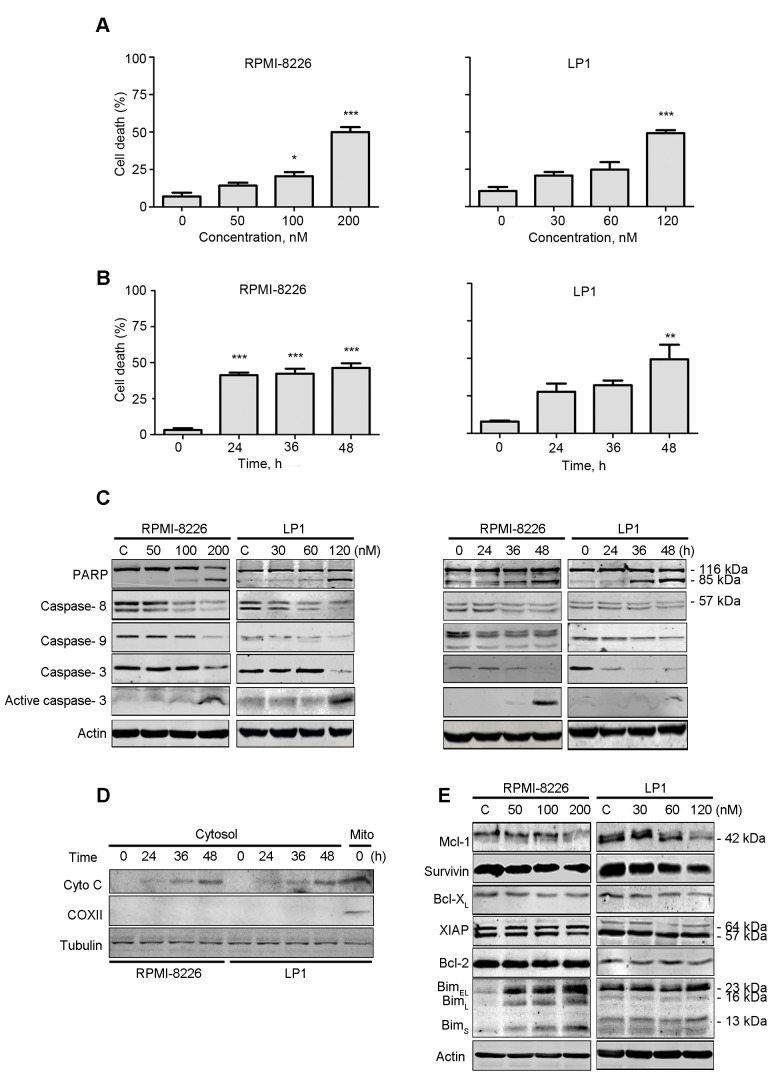
PP induces apoptosis in MM. RPMI-8226 and LP1 cells were treated with PP at (A) escalating concentrations for 48 h or (B) a fixed concentration (200 nM for RPMI-8226 and 120 nM for LP1) for different durations. A trypan blue exclusion assay was performed to count viable cells. (C) Western blotting analysis of RPMI-8226 and LP1 cells treated with PP for 48 h or at a fixed concentration (200 nM for RPMI-8226 and 120 nM for LP1) for different durations. (D) RPMI-8226 and LP1 cells were exposed to the indicated levels of PP for 48 h; the levels of cytochrome *c* in the cytosolic fractions were monitored by western blotting. COXII served as a mitochondrial indicator to exclude the contamination of cytosolic fraction by mitochondria. (E) Western blotting analysis of apoptosis-associated proteins in RPMI-8226 and LP1 cells upon treatment with PP. *P<0.05, **P<0.01, ***P<0.001; compared with the control. PP, pyrvinium pamoate; MM, multiple myeloma; Bcl-2, B-cell lymphoma 2; COXII, cyclooxygenase 2; BclXL, Bcl extra large; Bim, interacting mediator of cell death; XIAP, X-linked inhibitor of apoptosis protein; Mcl-1, myeloid cell leukemia 1.

